# Genome-wide association in *Drosophila* identifies a role for *Piezo* and *Proc-R* in sleep latency

**DOI:** 10.1038/s41598-023-50552-z

**Published:** 2024-01-02

**Authors:** Matthew N. Eiman, Shailesh Kumar, Yazmin L. Serrano Negron, Terry R. Tansey, Susan T. Harbison

**Affiliations:** 1 Laboratory of Systems Genetics, National Heart Lung and Blood Institute, National Institutes of Health, Bethesda, MD USA; 2 Present Address: Drexel University College of Medicine, Philadelphia, PA USA; 3 Present Address: Division of Neuroscience and Behavior, National Institute On Alcohol Abuse and Alcoholism, National Institutes of Health, Bethesda, MD USA

**Keywords:** Genetics, Behavioural genetics, Genetic association study, Heritable quantitative trait

## Abstract

Sleep latency, the amount of time that it takes an individual to fall asleep, is a key indicator of sleep need. Sleep latency varies considerably both among and within species and is heritable, but lacks a comprehensive description of its underlying genetic network. Here we conduct a genome-wide association study of sleep latency. Using previously collected sleep and activity data on a wild-derived population of flies, we calculate sleep latency, confirming significant, heritable genetic variation for this complex trait. We identify 520 polymorphisms in 248 genes contributing to variability in sleep latency. Tests of mutations in 23 candidate genes and additional putative pan-neuronal knockdown of 9 of them implicated *CG44153*, *Piezo*, *Proc-R* and *Rbp6* in sleep latency. Two large-effect mutations in the genes *Proc-R* and *Piezo* were further confirmed via genetic rescue. This work greatly enhances our understanding of the genetic factors that influence variation in sleep latency.

## Introduction

Sleep onset latency in humans is the number of minutes that it takes to fall asleep once the attempt to sleep is made. It is the difference between the desired sleep time and the actual start of sleep. Short sleep latencies may reflect sleep deprivation or excessive sleepiness^1^. Long sleep latencies have a more nuanced interpretation; they may reflect a reduced sleep need, or they may reflect difficulty in falling asleep due to increased arousal, as occurs in individuals with insomnia^2^. Researchers use a combination of polysomnography, actigraphy, and self-report/questionnaires to assess sleep latency in humans^1,3–7^. Human mean sleep latencies range from 10 to 21 min^3,4^. Heritabilities for human sleep latency are moderate, from 0.18 to 0.32^1,4^, suggesting that genes influence this trait. Accordingly, several human studies identified candidate genes for sleep latency. SNPs in the third intron of the CACNA1C gene were associated with sleep latency in individuals from the Australian Twin Registry^4^. A GWAS of sleep and activity parameters assessed with actigraphy found a SNP near DMRT1 associated with sleep latency^3^. A meta-analysis of GWAS found that sleep latency associated with three variants in an intron of RBFOX3^1^. Also, a scan of 2000 candidate genes identified polymorphisms in DRD2 associated with sleep latency and sleep duration^5^. In addition, candidate gene studies identified the 5-HTR2A receptor^6^ as being associated with sleep latency, and MTNR1B with REM sleep latency^7^. Thus, sleep latency exhibits a partial genetic basis in humans, and several candidate genes have been identified for the trait.

Additional evidence indicates that sleep latency is a complex trait, modifiable by potentially large numbers of genes. Sleep latency is often measured after a subject has been deprived of sleep. The earliest use of sleep latency as an objective measure of sleep debt noted a roughly linear relationship between the amount sleep lost and the corresponding sleep latency in humans^8^. However, the linear relationship could be altered by sampling sleep latency at different timepoints, suggesting the involvement of the molecular circadian clock^8^, and the relationship between sleep latency and the circadian clock was later demonstrated in flies^9^. The correlation between sleep latency and prior wakefulness has been observed in rodent models as well^10^, but can be disrupted by exposing the animals to different types of sleep-depriving stimuli^11^. For example, sleep latency was reduced in mice deprived of sleep via gentle handling but increased when constant cage change was used to deprive the animals of sleep, despite the fact that the amount of sleep lost was the same among groups^11^. Broad-sense heritabilities for sleep latency in mice under two different handling conditions were estimated to be between 0.28 and 0.70 for Diversity Outcross founder strains, comparable to heritabilities found in humans; however, narrow-sense (i.e., additive) heritability estimates for the Diversity Outcross itself were low, indicating a significant influence of both dominance and epistasis on this trait^12^. The overall implication is that while sleep latency has a genetic component, it can be perturbed by the circadian factors, sensory experience, and arousal in addition to exhibiting epistasis and dominance.

Sleep latency in flies is typically calculated without additional experimental intervention. Night sleep latency (often simply referred to as sleep latency) is the number of minutes that it takes the flies to fall asleep after the lights are turned off. Day sleep latency has a similar definition—it is the number of minutes it takes the fly to fall asleep after the lights are turned on. These quantitative measures in flies reflect the endogenous need to sleep. Though many mutant screens and candidate gene approaches report the effects of mutations on sleep latency (for example *wake*^9^, *Rdl*^13^, *amn*^14^, and *NPF* and *NPFR1*^15^), a systematic unbiased search for the genes that contribute to variability in night sleep latency in nature has not yet been conducted in flies. We therefore conducted a genome-wide association study of night sleep latency in the *Drosophila* Genetic Reference Panel (DGRP). The DGRP is a community resource created to identify genetic variants underlying complex traits like sleep latency^16,17^. The DGRP has been used to investigate over 61 complex traits^18^, including sleep, day-to-day fluctuations in sleep, and circadian behavior^19–21^. We found that sleep latency was not only variable in the DGRP, but heritable as well. Sleep latency is genetically correlated with many sleep traits, including a strong negative genetic correlation with night sleep duration. We mapped 520 variants tagging 248 genes to sleep latency, and verified candidate genes through mutational analysis and genetic rescue.

## Results

### Sleep latency is highly variable and heritable in the DGRP

**Figure 1:**
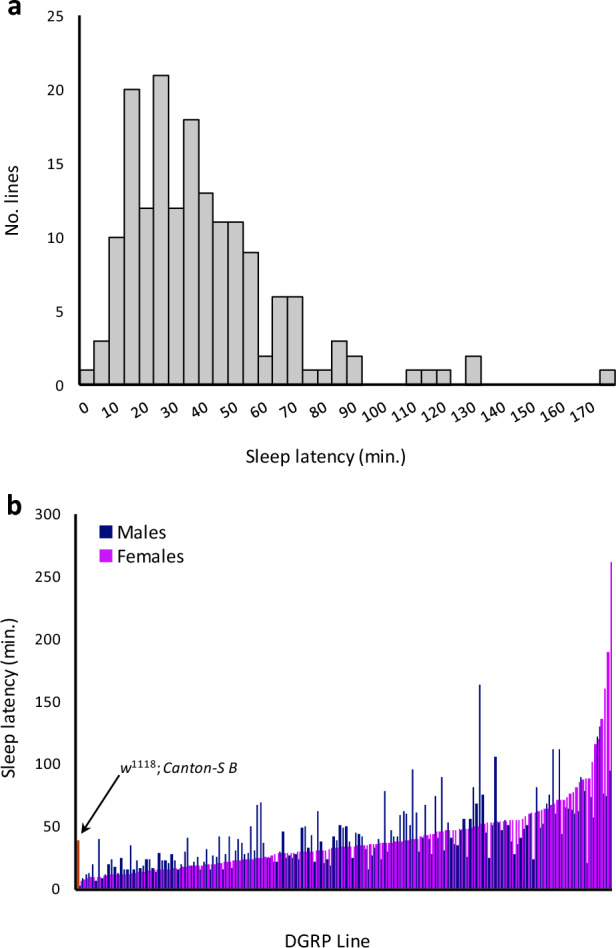
Sleep latency is highly variable and sexually dimorphic in the DGRP. (**a**), the histogram shows the distribution of sleep latency in the DGRP. (**b**), Mean sleep latency for each line/sex ordered numerically by sleep latency in females. *w*^1118^; *Canton-S B* control flies are plotted at the beginning of the distribution, with males shown by a light orange bar and females shown by a dark orange bar.

Mean sleep latency was highly variable in the DGRP, ranging from 4.2 min ± 5.2 SD to 176.6 min ± 148.7 SD, with a population average of 41.4 min ± 2.0 SD (Fig. 1A; Supplementary Table S1). Similar to the male population mean of 42.08 min ± 0.52 SD and the female population mean of 40.45 min ± 0.66 SD, *w*^1118^; *Canton-S B* control males had a sleep latency of 30.91 min ± 1.12 SD, while control females had a sleep latency of 39.26 min ± 2.11 SD. The genetic component of variance was highly significant for both sexes combined or for each sex separately (*P*_*Line(Block)*_ < 0.0001) (Table 1). Accordingly, the combined-sex broad-sense heritability *H*^2^ was 0.44, relatively high for a behavioral trait. Sleep latency was highly sexually dimorphic; consequently, the cross-sex genetic correlation was low (*r*_*mf*_ = 0.32), suggesting that sex-specific differences in the genetic basis of sleep latency exist. When mean sleep latencies are ordered numerically for females and compared to males, large differences between the two sexes can be seen (Fig. 1B). The minimum sleep latency in females was 6.2 min while the maximum was 261.7 min, a greater range than males, whose sleep latencies ranged from 2.1 to 163.3 min. The high levels of genetic variance and heritability for sleep latency indicated that genome-wide association mapping would be fruitful.

**Table 1:** Quantitative genetic analysis of sleep latency.

Sex	Source	*d.f*	M.S	F	*P*	*σ*^2^	*H*^2^	*r*_mf_
Combined	Block	3	52,165.08	1.20	0.3133	2.46	0.44	0.32
	Sex	1	6536.99	0.62	0.4338	Fixed		
	Line (Block)	164	40,502.69	3.99	< 0.0001	510.44		
	Sex × Line (Block)	164	9963.45	7.75	< 0.0001	289.05		
	Rep (Block)	12	4781.18	2.21	0.0803	4.37		
	Sex × Rep (Block)	12	1989.84	1.55	0.1038	1.86		
	Line × Rep (Block)	492	1459.89	1.13	0.0817	11.47		
	Sex × Line × Rep (Block)	490	1287.50	1.37	< 0.0001	45.47		
	Error	8897	941.28	–	–	941.22		
Males	Block	3	17,143.10	0.83	0.4810	0.00	0.45	
	Line (Block)	164	19,799.61	17.60	< 0.0001	617.69		
	Rep (Block)	12	2179.83	1.94	0.0281	3.35		
	Line × Rep (Block)	490	1126.91	1.58	< 0.0001	54.59		
	Error	4462	711.18	–	–	711.01		
Females	Block	3	42,328.98	1.27	0.2881	6.63	0.44	
	Line (Block)	164	30,669.99	18.95	< 0.0001	979.86		
	Rep (Block)	12	4545.23	2.81	0.0010	8.85		
	Line × Rep (Block)	492	1620.16	1.38	< 0.0001	58.92		
	Error	4435	1172.77	–	–	1172.89		

*d.f.*, Degrees of freedom; M.S., mean sum of squares; F, F-value; *P*, *P*-value; *σ*^2^, variance estimated by restricted maximum likelihood; *H*^2^, broad-sense heritability; *r*_mf_, cross-sex correlation.

### Genotype–phenotype associations identify candidate genes for sleep latency

**Figure 2:**
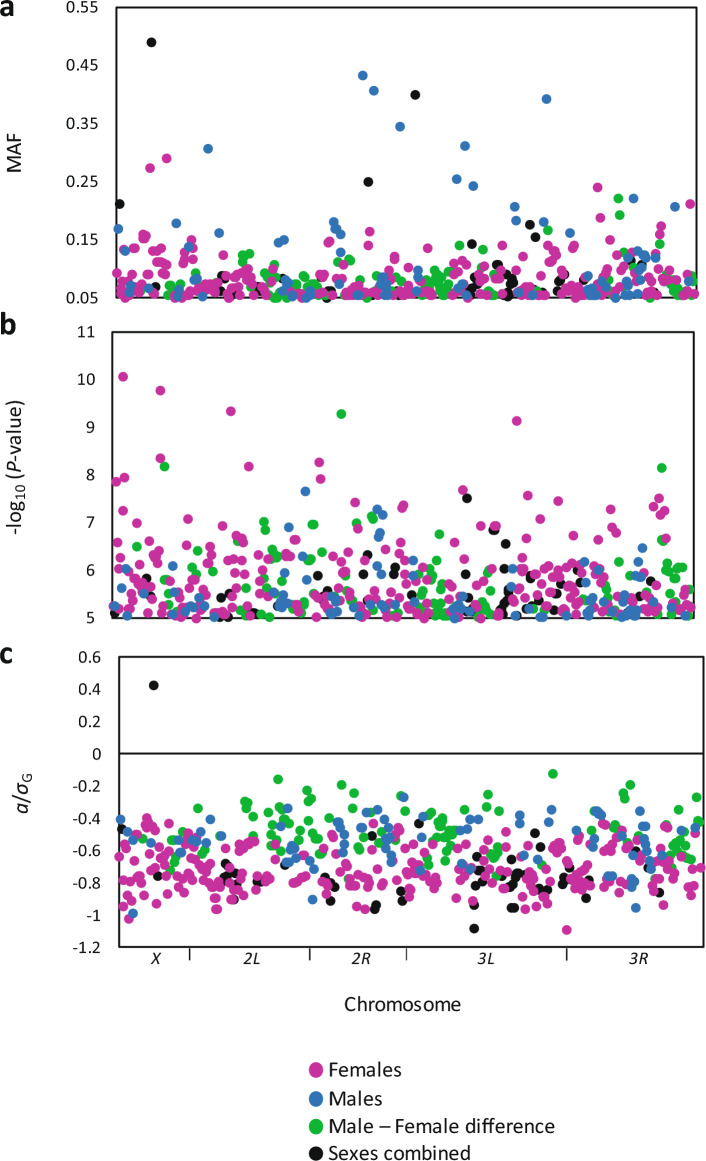
Multiple sex-specific loci are associated with sleep latency. The 520 polymorphisms significantly associated with sleep latency (*P* ≤ 1 × 10^–5^) are plotted. Each point is color coded to indicate the most significant association from the female-only, male-only, combined sexes, or sex difference analysis. (**a**), plot of minor allele frequency (MAF) versus genomic location. (**b**), plot of -log_10_(*P*-value) versus genomic location. (**c**), plot of effect size *a* normalized by the genetic standard deviation *σ*_G_.

The presence of genetic variance indicates that sleep latency can be mapped to the genome, but the low cross-sex genetic correlation suggests that different genes may contribute to sleep latency in males and females. Accordingly, we conducted the genome-wide association tests with male and female sleep latency separately and the difference between the line means of each sex (male–female) in addition to the analysis for both sexes combined. The DGRP2 webtool first determines whether *Wolbachia pipientis* infection and chromosomal inversions affect the phenotype of interest^17^, in this case sleep latency; however, neither *Wolbachia* nor inversion status had a significant effect. Using the DGRP2 webtool, we identified 520 unique single nucleotide polymorphisms (SNPs) that mapped to within ± 1000 bp of 248 genes (Fig. 2; Supplementary Table S2). For sexes combined, 132 polymorphisms mapped to 67 genes, with 168 polymorphisms mapping to intergenic regions. Most of the polymorphisms significant in the male-only analysis were unique to males; of the 95 polymorphisms mapping to male sleep latency, 17 overlapped with the sexes-combined analysis, only 2 overlapped with female sleep latency, and 2 overlapped with the sex difference analysis. In contrast, polymorphisms significant in the female-only analysis overlapped with 45% of the polymorphisms from the sexes-combined and sex difference analysis (146 out of 304). Minor allele frequencies were relatively low, with a mean 0.09 for all analyses (Fig. 2A). The relatively low minor allele frequencies of significant SNPs are typical of genome-wide association studies in sleep^19,20^ as well as other phenotypes in the DGRP^16,22–25^. A recent meta-analysis of phenotypes studied using the DGRP reported that lower-frequency alleles tend to have higher effect sizes and pleiotropic effects^26^. Little linkage disequilibrium existed among significant polymorphisms, also a typical characteristic of DGRP studies^19,20,22,24,25^. Combined-sex effect sizes, estimated as one-half the difference between the major and minor allele means, were large, ranging from − 24.8 to + 9.5 min per SNP, with an average of − 14.5 min. Interestingly, the presence of the minor allele was associated with an increase in sleep latency for all but one polymorphism, which mapped to an intergenic region of the *X* chromosome (Fig. 2C). The effects of the more common alleles in this population, therefore, were in the direction of reduced sleep latency. Thus, sleep latency is a typical complex trait influenced by many genes, some of which have sex-specific effects.

Several of the 248 candidate genes were enriched for certain KEGG pathways and Gene Ontology categories. Seven Hippo pathway genes were implicated in sleep latency: *app*, *CycE*, *dlg1*, *Dlg5*, *ed*, *fred*, and *scrib* (FDR = 0.0139). Enrichment was also present for several biological process categories: synaptic target recognition (FDR = 0.0200), motor neuron axon guidance (FDR = 0.0228), homophilic cell–cell adhesion via plasma membrane adhesion molecules (FDR = 0.0013), and cell–cell adhesion (FDR = 0.0463) (Supplementary Table S3). The list was also enriched for plasma membrane proteins (FDR = 0.0013). We used DIOPT (DRSC Integrative Ortholog Prediction Tool) to identify human genes with high homology to the fly genes associated with sleep latency^27^. While 44 of the genes had no known human orthologs, 136 genes (54.8%) had high homology with at least one human gene, and 65 (26.2%) had more than one predicted human homolog (the remaining 3 genes were not found in the database) (Supplementary Table S4). Thus, the vast majority of the genes we identified, 81%, had human orthologs.

We also examined the phenotypic and genetic correlations between sleep latency and other sleep and circadian traits (Table 2). Notably, night sleep duration had a very strong negative correlation with sleep latency (*r*_G_ = -1.0, *P* ≤ 0.0001), implying similar genetic architecture between night sleep duration and sleep latency. Overlapping the SNPs we previously identified for night sleep duration with sleep latency SNPs revealed three overlapping SNPs and two overlapping candidate genes, *Rbp6* and *alpha-Man-I* (Supplementary Tables S5 and S6)^19^. In addition, seven genes (*CG32103*, *CG34353*, *fz*, *kirre*, *mam*, *MsR1*, and *scrib*) that we identified for night sleep duration from an artificial selection experiment also overlapped with sleep latency genes^28^. Tests of a mutation in *fz* in that study revealed a suggestive pleiotropic effect on sleep latency, increasing it in females^28^. Like night sleep duration, a high correlation [*r*_G_ = 1.0 (*P* ≤ 0.0001)] existed between sleep latency and night sleep duration *CV*_E_ (the coefficient of environmental variation). Night sleep duration *CV*_E_ is a measure of the variability in sleep duration observed between flies with the same genotype^19^. Forty-seven SNPs overlapped between sleep latency and night sleep duration *CV*_E_, and twenty genes overlapped (Supplementary Tables S5 and S6)^19^. In addition, one intergenic SNP overlapped between sleep latency and night sleep σ, which measures the day-to-day fluctuations in night sleep in each fly^20^. Finally, one intergenic SNP overlapped between and sleep latency and rhythmicity index, the degree of similarity among daily activity patterns^21^. The shared genetic architecture between sleep latency and other sleep and circadian traits implies pleiotropic gene effects.

**Table 2:** Phenotypic and genetic correlations between sleep latency and other sleep and circadian traits.

Trait	*r*_P_	*r*_G_
Night sleep (min.)	**− 0.7726**	**− 1.0000**
Night avg. bout length (min.)	**− 0.3733**	**− 0.5914**
Day sleep (min.)	**− 0.2270**	**− 0.4251**
Day avg. bout length (min.)	**− 0.1079**	**− 0.2785**
Waking activity *CV*_E_	**− 0.1486**	**− 0.2655**
Night bout number *CV*_E_	**−** 0.1022	**− 0.2053**
Day bout number	**−** 0.0980	**− 0.1818**
Day avg. bout length *CV*_E_	**−** 0.0343	**−** 0.1040
Rhythmicity index	0.0098	0.0005
*Χ*^2^ period	0.0182	0.0008
MESA period	0.0135	0.0009
Waking activity (cts/min)	0.0242	0.0013
Night avg bout length *CV*_E_	0.0692	**0.1666**
Night bout number	**0.2153**	**0.3433**
Day bout number *CV*_E_	**0.1337**	**0.3488**
Day sleep *CV*_E_	**0.1913**	**0.4203**
Night sleep *CV*_E_	**0.7944**	**1.0000**

*r*_G_, Genetic correlation; *r*_P_, phenotypic correlation; *CV*_E_, coefficient of environmental variation. Numbers in bold are statistically significant.

In addition, we tested 43 SNPs with moderate-to-high minor allele frequencies for potential SNP-SNP interactions (i.e., epistasis). We found 6 significant pairwise interactions in males, and 100 in females (Supplementary Table S7), providing evidence that epistasis contributes to sleep latency.

**Figure 3:**
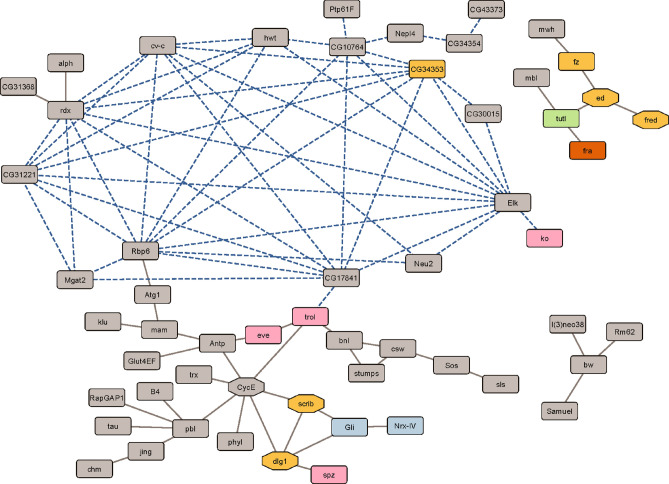
Interaction network of sleep latency genes. Known genetic and protein–protein interactions between genes via BIOGRID are plotted as solid gray lines. Epistatic interactions are plotted as blue dashed lines. Genes involved in synaptic target recognition are plotted in blue. Genes that have functions in motor neuron axon guidance are plotted in pink. Genes that are involved in cell–cell adhesion, including homophilic cell adhesion, are plotted in light orange. Gene names within octagons are part of the Hippo pathway. *Tutl*, which has known functions in synaptic target recognition and cell–cell adhesion, is plotted in green. *Fra*, which has known functions in motor neuron axon guidance and cell–cell adhesion, is plotted in dark orange. Only networks with connections among four or more genes were plotted; for the full list of genes, see Supplementary Table S8.

We combined the data from the epistasis analysis and the Gene Ontology analysis in the following way. We searched the BIOGRID^29^ database for evidence of genetic and protein–protein interactions among sleep latency candidate genes. We found 82 interactions among 69 sleep latency genes. We combined the BIOGRID data with our epistasis analysis, where the epistatic SNPs mapped to within ± 1000 bp of a gene. Figure 3 shows the resulting network, with Gene Ontology categories and gene pathways annotated (see Supplementary Table S8 for the list of genes and annotations). Interestingly, the epistatic network connects to the known genetic and protein–protein interaction network via three genes: *RNA-binding protein 6* (*Rbp6*), *terribly reduced optic lobes* (*trol*), and *roadkill* (*rdx*).

### Mutant and RNAi knockdown tests verify candidate genes for sleep latency

**Figure 4:**
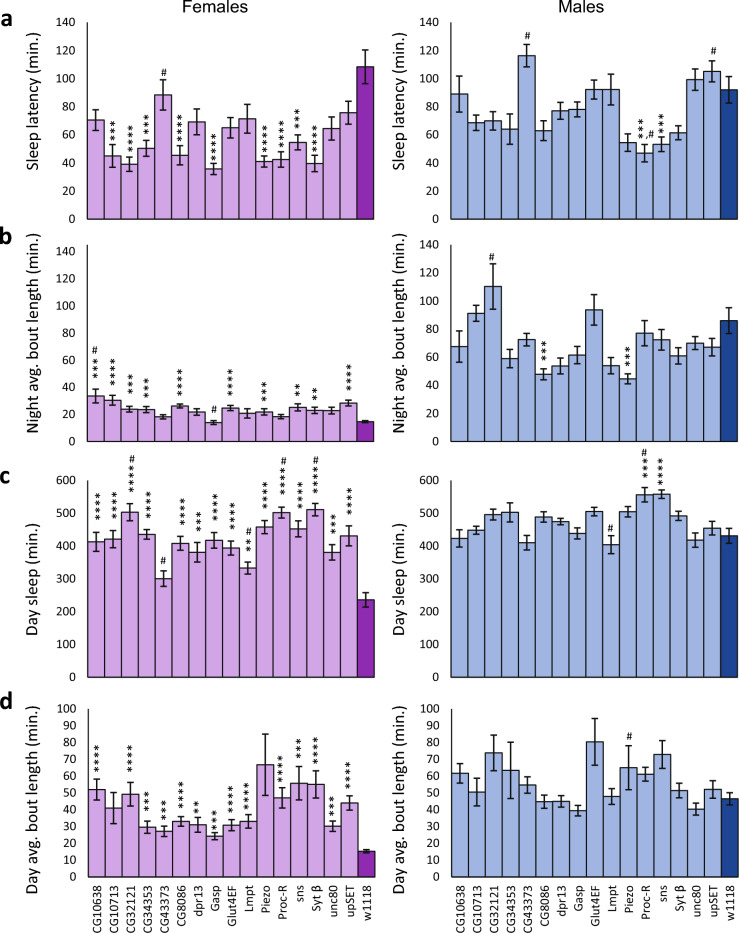
*Minos* insertions having a *w*^1118^ control affect sleep latency and exhibit pleiotropic effects on some sleep phenotypes. The plots show mean sleep phenotypes ± SEM in *Minos* insertion lines contrasted with the sleep phenotypes of the *w*^1118^ isogenic control. Light purple bars indicate female data, with the *w*^1118^ control shown in dark purple. Light blue bars indicate male data, with the *w*^1118^ control shown in dark blue. (**a**) Sleep latency. (**b**) Night avg. bout length. (**c**) Day sleep duration. (**d**) Day avg. bout length. Asterisks show the level of significance: **** *P* ≤ 0.0001; *** 0.0001 < *P* ≤ 0.001; ** 0.001 < *P* ≤ 0.0031. A pound sign (#) shows those mutants that are also significantly different from the overall mean of all mutant data combined.

Using the genome-wide association data, we chose candidate genes to verify. We selected the 100 polymorphisms having the largest effect sizes for both sexes combined that mapped to within ± 1000 bp of a gene. Twenty-three of these genes had *Minos* insertion lines available for testing. These transposable element insertions putatively disrupt gene function^30^. We measured sleep and activity in these mutants, and compared them with their isogenic controls. To analyze the mutant data, we considered both sexes-combined data and we also analyzed the data for each sex separately (Supplementary Table S9). For both sexes combined, sleep latency decreased significantly in comparison to the *w*^1118^ control for nine *Minos* mutations (Bonferroni *P*-value < 0.0031), including *CG10713*, *CG32121*, *CG34353*, *CG8086*, *Gasp*, *Piezo*, *Proc-R*, *sns*, and *Syt β*. Significant decreases in sleep latency were present in females for all nine mutations as well (Fig. 4A). In males, there was a significant decrease in sleep latency for *Proc-R* and *sns* only, although the same trend of decreased sleep latency relative to the control was observed for all mutations (Fig. 4A). To mitigate any effect of the *w*^1118^ control line genetic background we additionally tested whether these mutants were significantly different from their overall mean using Dunnett’s *t*-test (*P* < 0.05). *CG43373*, *Piezo*, *Proc-R*, and *upSET* were significantly different from the overall mean for both sexes combined; *CG43373* was significantly different for both sexes separately; and *Proc-R* was significant for males (Supplementary Table S9). Both tests implicated *Piezo* and *Proc-R*, which had the most extreme sleep latencies.

Given the strong negative correlation between sleep latency and night sleep, night avg. bout length, day sleep, and day avg. bout length, one might anticipate pleiotropic effects on these traits. For females, we did observe increases in night avg. bout length, day sleep, and day avg. bout length in addition to the reduced sleep latency (Fig. 4B–D). For males, there was a corresponding increase in day sleep for *Proc-R* and *sns* (Fig. 4C). Little evidence of pleiotropy was present for night sleep or night bout number (Supplementary Fig. S2A,B) in either males or females. Likewise, few mutations had effects on day bout number or waking activity, as expected due to their low genetic correlation with sleep latency (Supplementary Fig. S2C,D).

**Figure 5:**
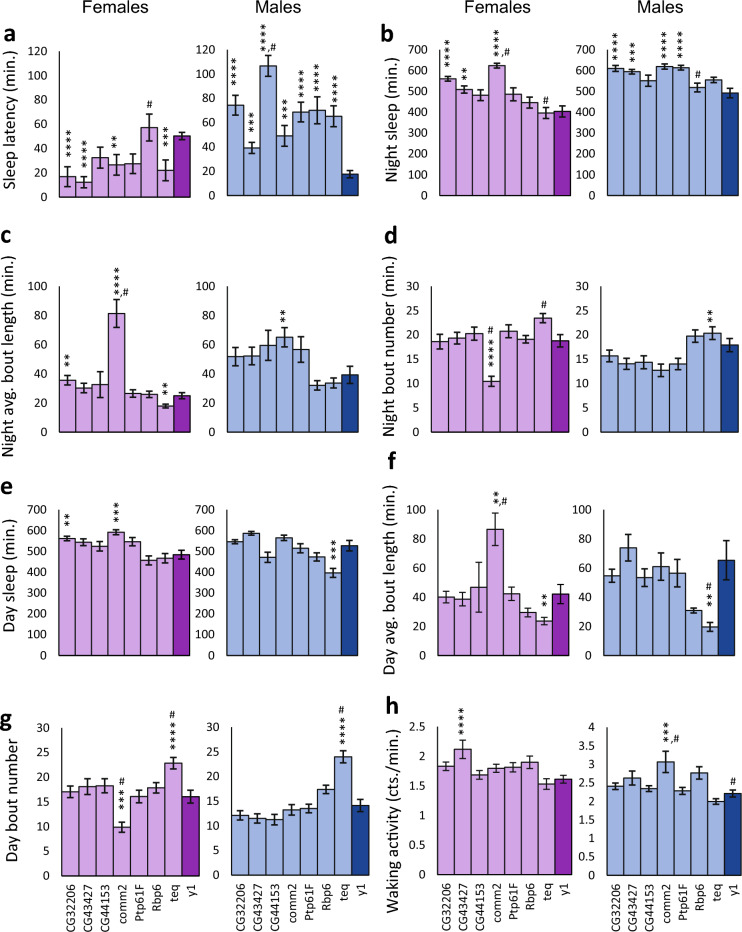
*Minos* insertion lines with *y*^1^*w*^67c23^ control affect sleep latency and exhibit pleiotropic effects on some sleep phenotypes. The plots show mean sleep phenotypes ± SEM in *Minos* insertion lines contrasted with the sleep phenotypes of the *y*^**1**^*w*^**67c23**^ isogenic control. Light purple bars indicate female data, with the *y*^**1**^*w*^**67c23**^ control shown in dark purple. Light blue bars indicate male data, with the *w*^1118^ control shown in dark blue. (**a**) Sleep latency. (**b**) Night sleep duration. (**c**) Night average bout length. (**d**) Night bout number. (**e**) Day sleep. (**f**) Day average bout length. (**g**) Day bout number. (**h**) Waking activity. Asterisks show the level of significance: **** *P* ≤ 0.0001; *** 0.0001 < *P* ≤ 0.001; ** 0.001 < *P* ≤ 0.0071. A pound sign (#) shows those mutants that are also significantly different from the overall mean of all mutant data combined.

Unlike the lines with the *w*^1118^ control, the direction of effects of *Minos* insertions in the *y*^1^*w*^67c23^ background were highly sex dimorphic. Four lines with insertions in the genes *CG32206*, *CG43427*, *comm2*, and *teq* had decreased sleep latency in females relative to the control (Fig. 5A; Bonferroni *P*-value < 0.0071; Supplementary Table S9). In contrast, all seven *Minos* insertions had significant changes in sleep latency in males—but sleep latency was increased rather than decreased relative to the control (Fig. 5A). In females, little pleiotropy was observed in the other sleep traits (Fig. 5B–H). A *Minos* insertion in one gene, *comm2*, exhibited the most pleiotropy, affecting not only sleep latency but night and day sleep duration, avg. bout length, and bout number (Fig. 5B–G). *teq* was highly pleiotropic in males, with changes occurring in night bout number, day sleep duration, day avg. bout length, and day bout number (Fig. 5D–G). For four of the *Minos* insertions, there was an increase in sleep latency in males, and a corresponding increase in night sleep, contrary to the expected negative correlation between these variables (Fig. 5A,B). Again we tested whether there were differences in sleep latency between these mutations and their overall mean using Dunnett’s *t*-test (*P* < 0.05) to mitigate any *y*^1^*w*^67c23^ control line effects. *CG43427*, *CG44153*, and *Rbp6* were significant for sexes combined; *CG44153* was significant for males, and *Rbp6* was significant for females. Overall, 16 of the 23 *Minos* mutants tested had effects on sleep latency exceeding a strict Bonferroni correction in at least one sex, and the additional statistical tests supported *Piezo*, *Proc-R*, *CG43427*, *CG44153*, and *Rbp6* as candidate genes for sleep latency.

**Figure 6:**
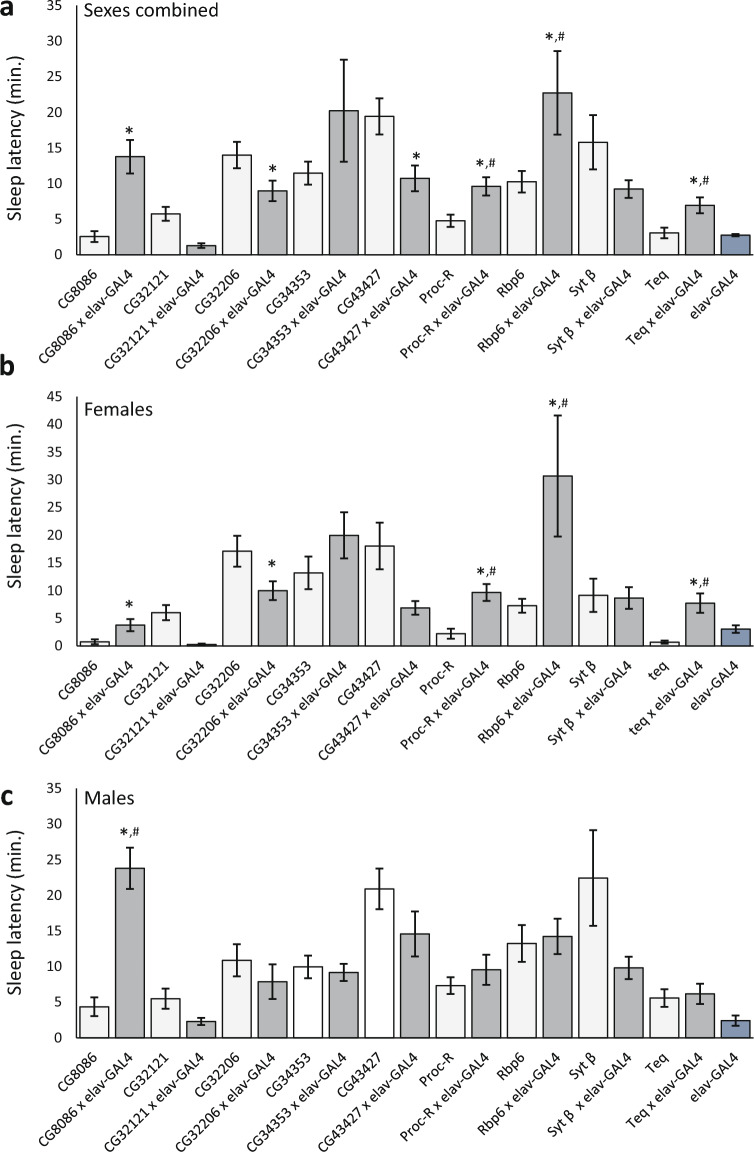
Sleep latency in TriP RNAi knockdown lines. The plots show sleep latency for each RNAi line, the *elav*-GAL4 pan-neuronal driver, and the *elav*-GAL4 × RNAi cross. (**a**) Sexes combined; (**b**) Females; (**c**) Males. An asterisk (*) is plotted for each cross that is significantly different from both the *elav-GAL4* driver and its respective RNAi line. A pound sign (#) shows those crosses that are also significantly different from strict additivity.

We used an additional approach to test nine of the candidate genes having significant *Minos* insertion sleep latency phenotypes. We used RNAi to knock down the following genes pan-neuronally with an *elav*-GAL4 driver: *CG32121*, *CG32206*, *CG34353*, *CG43427*, *CG8086*, *Proc-R*, *Rbp6*, *Syt-β*, and *teq*. We compared each *elav*-GAL4 × RNAi cross with its respective RNAi and the *elav*-GAL4 driver line as controls. Sleep latency in each cross had a significant effect on genotype for sexes combined (Fig. 6A; Bonferroni-corrected *P*-value = 0.0055; Supplementary Table S10). We then examined the data for each sex separately (Fig. 6B,C). *CG32121*, *CG32206*, *CG43427*, *Proc-R*, and *teq* had significant genotypic effects in females, and *CG8086*, *CG34353*, *CG43427*, and *Rbp6* had significant genotypic effects in males (Bonferroni-corrected *P*-value = 0.0055; Supplementary Table S10). Post-hoc Tukey analysis on the combined-sex data revealed that all but three of the *elav*-GAL4 × RNAi crosses (*CG32121*, *CG34353*, and *Syt β*) were significantly different from both the corresponding RNAi line as well as the *elav*-GAL4 driver. For females, *CG32206*, *CG8086*, *Proc-R*, *Rbp6*, and *teq elav*-GAL4 × RNAi crosses were significantly different from all controls; for males, only *CG8086* crosses were significantly different (Fig. 6B,C). Inspection of Fig. 6 reveals that flies from the *elav*-GAL4 × RNAi cross have sleep latencies that are intermediate between the controls and could be due to the heterozygous background. We tested this formally using a t-test to determine whether the sleep phenotypes in each *elav*-GAL4 × RNAi cross were different from what would be expected under a strictly additive model. For females and both sexes combined, *Proc-R*, *Rbp6*, and *teq* crosses were both significantly different from controls and significantly different from an additive model, while males of the *CG8086* cross were significantly different from controls and significantly different from an additive model (Supplementary Table S10). Thus, pan-neuronal RNAi knockdown corroborated the role of *Proc-R* and *Rbp6* in sleep latency.

Overall, the combined mutational and RNAi analysis suggested that *CG44153*, *Piezo*, *Proc-R*, and *Rbp6* are candidate genes for sleep latency.

### Precise excisions rescue sleep latency phenotypes in *Piezo* and *Proc-R* mutants

**Figure 7:**
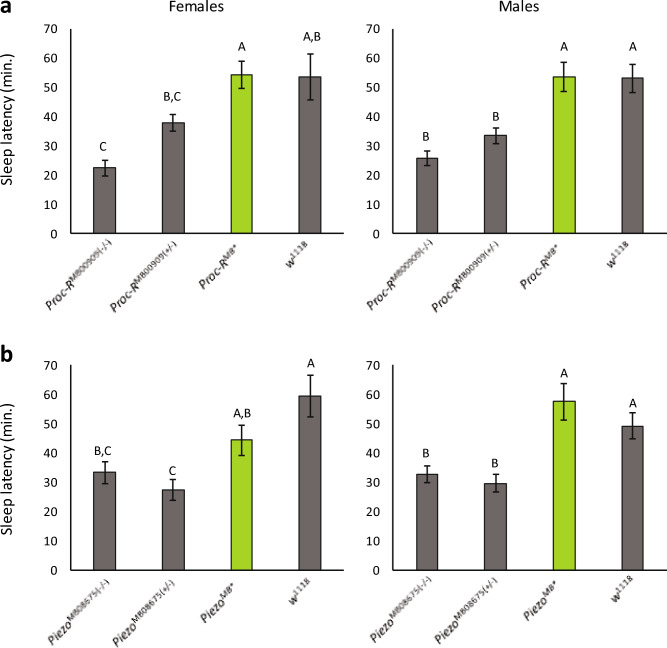
Precise excision of *Minos* insertions in *Piezo* and *Proc-R* rescues wildtype sleep latency. The figures plot mean sleep latency for each line ± SEM. The rescued line (MB^*^) is marked in green. Means having the same letter are not significantly different by post-hoc Tukey test. (**a**) *Piezo* precise excision data for females and males. For females, sleep latency in the *w*^1118^ control was 59.39 ± 7.0 min (n = 32); *Piezo*^MB*^ (precise excision) was 44.39 ± 5.2 min (n = 32); *Piezo*^MB08675^ heterozygote was 27.46 ± 3.4 min (n = 30); and *Piezo*^MB08675^ homozygous mutant was 33.45 ± 3.7 min (n = 31). For males, sleep latency in the *w*^1118^ control was 49.31 ± 7.0 min (n = 32); *Piezo*^MB*^ precise excision was 57.54 ± 6.1 min (n = 32); *Piezo*^MB08675^ heterozygote was 29.68 ± 3.0 min (n = 30); and *Piezo*^MB08675^ homozygous mutant was 32.77 ± 2.9 min (n = 32). (**b**) *Proc-R* precise excision for females and males. For females, sleep latency in the *w*^1118^ control was 53.48 ± 7.9 min (n = 32); *Proc-R*^MB*^ (precise excision) was 54.22 ± 4.7 min (n = 32); *Proc-R*^MB00909^ heterozygote was 38.02 ± 2.9 min (n = 31); and *Proc-R*^MB00909^ homozygous mutant was 22.39 ± 2.6 min (n = 32). For males, sleep latency in the *w*^1118^ control was 53.00 ± 4.7 min (n = 32); *Proc-R*^MB*^ was 53.51 ± 5.2 min (n = 29); *Proc-R*^MB00909^ heterozygote was 33.48 ± 2.7 min (n = 31); and *Proc-R*^MB00909^ homozygous mutant was 25.74 ± 2.4 min (n = 31).

We created precise excision lines for *Piezo* and *Proc-R* as the *Minos* insertions in these two genes had the most extreme decrease in sleep latency for both sexes combined compared to the *w*^1118^ control (Fig. 7A; Supplementary Table S9; Supplementary Figs. S3 and S4). Sleep in *Proc-R* and *Piezo* mutants occurred 55.3 and 52.3 min before that of the *w*^1118^ control, respectively. For each gene, we tested the sleep latency in the homozygous *Minos* insertion line, the *w*^1118^ control, and a heterozygous cross of the *Minos* insertion line and control against the precise excision line. The precise excision in *Proc-R* completely rescued the wildtype phenotype in both females and males (Fig. 7A). The precise excision line for *Piezo* partially rescued the wildtype phenotype in females and completely rescued the wildtype phenotype in males. For both sexes, sleep latency in the precise excision was indistinguishable from the *w*^1118^ control (Fig. 7B). However, for females, sleep latency in the precise excision was also not significantly different from the homozygous *Piezo Minos* insertion line while for males there was a clear distinction between the precise excision and the homozygous and heterozygous *Piezo Minos* insertion line. Sleep latency thus maps to both of these candidate genes. In addition, given its role in detecting mechanical stimuli^31^, we wondered whether *Piezo* mutants would respond to mechanical shaking during sleep. We measured the arousal threshold of *Piezo* mutants by stimulating them mechanically once per hour for three days. In comparison to *w*^1118^ control and precise excision flies, the proportion of *Piezo* mutant flies responding to the mechanical stimulus was the same, day or night (Supplementary Fig. S5). However, *Piezo* mutants slept longer prior to the stimulus, and were awake for less time after the stimulus during the night.

## Discussion

Here we conducted a genome-wide association for sleep latency in flies, greatly extending the catalog of known variants and genes that modify this trait. Sleep latency was both genetically variable and heritable, and interestingly, had a greater range in females than males of the DGRP. We found a strong negative correlation between night sleep duration and sleep latency, consistent with previous artificial selection and candidate gene/mutagenesis experiments. Genetic mapping revealed 520 variants that mapped to 248 genes. By making use of the accessible *Minos* insertion lines, we effectively validated genes that were selected for testing their involvement in sleep latency. Moreover, our results were further confirmed by conducting experiments that utilized pan-neuronal knockdown through the *elav*-GAL4 driver. Notably, knockdown of *CG44153*, *Piezo*, *Proc-R*, and *Rbp6* provided supplementary evidence of their impact on sleep latency. Of particular significance, the mutations in the *Proc-R* and *Piezo* genes exhibited the most substantial influence on sleep latency; interestingly, we achieved a successful restoration of normal effects for both of these genes by precisely eliminating the *Minos* element.

Variants from the combined-sex and female-specific GWAS tended to overlap, but most of the male-specific variants were unique. We chose genes with larger predicted effect sizes from the combined-sex analysis for verification via mutational analyses; most of these were implicated in the female-specific analysis as well. Interestingly, *Minos* insertion mutants and TRiP RNAi knockdown also had stronger effects on both sexes combined and on females in particular than on males, despite the differences in genetic background between the DGRP, the *Minos* insertion lines, and the TRiP RNAi lines. This observation coupled with the low cross-sex correlation for sleep latency implies that different sets of genes influence sleep latency in males and females.

Sleep latency genes were connected to one another in a network using previously reported genetic/protein–protein interactions and significant epistatic interactions from our own analysis. The epistasis analysis we conducted implies a genetic interaction network in addition to known gene interactions, with three genes connecting the two networks: *Rbp6*, *trol*, and *rdx*. *Rbp6* is a RNA-binding protein implicated as a heterogeneous nuclear ribonucleoprotein with a putative role in the regulation of translation^32,33^. *trol* functions in many processes, including the regulation of cell signaling in hedgehog, FGF, and EGF pathways and the regulation of the stability of the extracellular matrix covering neurons^33,34^. *rdx* is part of the hedgehog signaling pathway, with a role in myriad developmental processes^35,36^. These three genes are highly pleiotropic, and the results from other genome-wide association studies using the DGRP support this observation. All three genes are implicated in olfactory avoidance and the resistance to oxidative stress^25,37^. *Rbp6* is a candidate for activity in response to a rotating stimulus^38^, fitness traits including age-specific fecundity and the influence of the female nervous system on sperm competition^39,40^; aggression^41^, alcohol sensitivity^42^, and infection resistance^43^. Further, the three genes are candidate genes for sleep and activity phenotypes: *Rbp6* for night sleep duration, night sleep *CV*_E_, and waking activity *CV*_E_; *rdx* for waking activity; and *trol* for day avg. bout length, night sleep *CV*_E_, and waking activity *CV*_E_^19^. Interestingly, *Rbp6* was also implicated in a GWAS of circadian period^21^, and its expression is down-regulated in a DGRP line with a long circadian period^44^. The high degree of pleiotropy in these three genes supports the omnigenic model of inheritance^45^, which suggests that most of the genetic variation in a trait can be attributed to genes having indirect effects.

Sleep latency tests of *Minos* insertions were uniformly decreased in flies compared to the *w*^1118^ genetic background, while they were they were mostly uniformly decreased in females and increased in males compared to the *y*^1^*w*^67c23^ genetic background. As we demonstrate here, many genomic variants may impact sleep latency; thus, one possibility is that there are de novo mutations in addition to the focal gene segregating in the genetic backgrounds of the *Minos* lines that enhance the effect on sleep latency in either the insertion line or the control. However, several lines of evidence argue against the idea that de novo mutations enhance or exaggerate sleep phenotypes. First, standing genetic variation for sleep and activity traits in the DGRP is much lower than would be expected compared to a neutrally evolving model, suggesting that stabilizing selection acts against de novo mutations to favor intermediate phenotypes over extremes^46^. Similarly, a selective breeding experiment demonstrated that natural selection acts against extreme long and short sleep duration, shifting the allele frequencies of genomic modifiers to generate more moderate sleep^47^. In addition, long-standing stocks of *Shaker* mutant alleles did not exhibit a short-sleeping phenotype until outcrossed, suggesting that accumulated de novo mutations suppressed the expected short sleep duration phenotype^48^. In contrast, outcrossing a mutation in the gene *Calreticulin* to the DGRP both suppressed and enhanced the effects of the mutation on sleep duration^49^. These data would suggest that selective forces would tend to suppress the effects of de novo mutations accumulating in the *Minos* lines. To further support the *Minos* data, we conducted additional tests using RNAi knockdown lines that were available for nine of the genes we tested. Combined testing and analysis suggested that *CG44153*, *Piezo*, *Proc-R*, and *Rbp6* impact sleep latency. We note that the effects of the *Minos* insertions are not an exact phenocopy of the effects observed using RNAi knockdown for *Proc-R* and *Rbp6*. The difference may be due to the location of the *Minos* insertion, or due to differences in genetic background^50^. A future direction would be to compare both types of knockdown in the same genetic background, including the *Minos* rescue experiments.

We conducted genetic rescue in *Proc-R*, suggesting that it has a role in sleep latency. *Proc-R* is the receptor for proctolin, a neuropeptide involved in the stimulation of muscle contraction in insects^51^. *Proc-R* expression was detected in glial and muscle cells using single-cell RNA-Seq^52^, while immunolabeling detected *Proc-R* in neurons of the brain and muscles of the hindgut^53^. *Proc-R* is expressed during all developmental stages and at the adult stage, with greater expression during early embryonic development^54^. Notably, *Proc-R* expression does not fluctuate in a circadian fashion; instead, it appears to be consistent across time and under different lighting conditions^53^. While a CRISPR/Cas9-induced knockout of *Proc-R* was assayed for sleep, no significant effect on day, night, or 24-h sleep was noted^55^, in contrast to the changes we observed in sleep duration. The identification of *Proc-R* as a candidate gene for sleep latency is intriguing given that one of the hallmarks of sleep is a relaxed muscle tone.

We also demonstrated that sleep latency maps to *Piezo*. *Piezo* is an ion channel expressed in sensory neurons that responds to both mechanical pressure and voltage^52,56,57^. Its expression is highest during embryonic development but it is expressed at all developmental stages, including the adult stage^54^. *Piezo* has many different functions in flies, including, for example, an intriguing role in axon regeneration after injury^58^. Knockouts of *Piezo* in larvae have a reduced response to strong mechanical stimuli mediated through *ppk*-positive neurons that line the larval body wall^31^. Responses to temperature change and gentle touch are normal in *Piezo* knockouts, suggesting that the channel detects only relatively strong mechanical stimuli in larvae^31^. Thus, the role of the *Piezo* polymorphism in sleep latency may involve an attenuation of arousal threshold during sleep. We tested this idea by measuring arousal threshold in *Piezo* mutants and their corresponding controls. While the proportion of sleeping flies responding to a repeated shaking stimulus during the night did not differ between *Piezo* mutants and controls, *Piezo* mutants slept more between each stimulus and stayed awake for less time after the stimulus, suggesting that they were less perturbed by the shaking. The reduced sleep latency of *Piezo* mutants may therefore reflect a dampened sensitivity to the environment, particularly mechanical stimuli in the environment. *Piezo* potentially influences sleep latency through satiety. Recent work demonstrates that dietary protein promotes sleep through peptidergic signaling in the posterior gut, specifically inhibiting the response to mechanical stimulation^59^. It would be interesting to determine whether *Piezo*, which functions to sense fullness in the anterior gut^60^, has a role in this signaling pathway.

The very high negative genetic correlation estimate between sleep duration and sleep latency that we calculated is consistent with much of the sleep literature in flies. For example, selective breeding for an insomnia-like phenotype and for short night sleep duration resulted in a correlated response for sleep latency, increasing it relative to control populations^28,61^. Similarly, selection for long night sleep had reduced sleep latency as a correlated response^28^. Likewise, mutational studies of genes affecting sleep duration reflect the negative correlation with sleep latency. For example, a reduction in night (and sometimes 24-h) sleep duration accompanied an increase in sleep latency for mutations in the following genes: *5-HT1A*, *5-HT1B*, *aus*, *BomBc2*, *cv-c* (also identified in this study), *ltpr*, *Lztr1*, *Nlg4*, *rogdi*, *Shal*, *tara*, *Trh*, and *wake*^9,62–71^. Overexpression of the genes *Appl* and *fbx14* revealed the same pattern^72,73^. Mutations that increase night sleep duration, such as those in *Ih*, *fbx14*, *Gabat*, *miR-276a*, *NPF*, *NPFR*, *para*, and *Rdl* have a corresponding decrease in sleep latency^13,15,72,74–77^. Interestingly, increasing *brp* synaptic protein levels extended night sleep and decreased sleep latency in a dose-dependent manner^78^. Previous work also observed a similar pattern for sleep during the day: increased day sleep latency accompanies reduced day sleep duration, as observed in mutants of *CG2277* and *tau* (also identified in this study)^79,80^. Mutants that increase day sleep duration have reduced sleep latency^75,79,81^. Only a few exceptions to this generalized result exist thus far: over-expression of *miR-375* in *tim*-expressing neurons decreased both sleep and sleep latency; heterozygous mutations in conserved exons of *wake* increased 24-h sleep duration and day sleep latency in females; and changes in sleep latency emerged without a corresponding change in sleep duration in pan-neuronal knockdowns of *CG32459* and *CG1814*^68,79,82^. The negative relationship between sleep duration and sleep latency persists across brain structures and is present when genes are overexpressed pan-neuronally^9,73^ and in large ventral lateral neurons^72^; and when genes are reduced pan-neuronally^65,79^, in small and large ventral lateral neurons^9,66,71,72,81^, in R5 neurons^83^, and in glia^63^. Much of the data from mutational studies in flies, therefore, supports a negative genetic correlation between sleep duration and sleep latency, and the identification of a gene affecting sleep latency apart from sleep duration is rare. Very long sleep duration is necessarily coupled with short sleep latency, as the longer a fly sleeps during the day or night, the less time there is available to spend falling asleep. However, why very short sleep duration also correlates with very long sleep latency in flies is not clear. One possible explanation would be a strong genetic correlation between sleep latency and the genetic components of the circadian clock, which could alter the timing of sleep. We did not observe any correlation or connection between sleep latency and circadian period or rhythmicity index except for a single SNP in an intron of *Pdp1* implicated for sleep latency in female flies.

Studying the genetics and environmental factors that influence sleep latency can offer valuable insights into the factors that contribute to insomnia and lead to the identification of novel therapeutic targets for the disorder. Here we have demonstrated significant genetic variation for sleep latency in a natural population of flies. In addition to sharing genetic architecture with night sleep duration, most of the genes mapping to this moderately heritable trait have human homologs. A long-term goal would be to determine whether the genes we have identified for sleep latency in flies are functionally conserved in humans.

## Materials and methods

### Drosophila stocks

This study re-analyzes sleep and activity data measured in a previous study of 167 lines of the *Drosophila* Genetic Reference Panel and *w*^1118^; *Canton-S B*^19^ in order to calculate the sleep latencies for each fly measured. We also tested 23 *Minos* insertion lines for potential effects on sleep latency (Bloomington *Drosophila* Stock Center, Bloomington, IN). Supplementary Table S9 lists the *Minos* lines, genotypes, Bloomington stock numbers, and control lines used. For the genetic rescue of *Piezo* and *Proc-R* mutants, we used *w*^1118^; *sna*^Sco^/SM6a, *P*[*w*[+ mC] = hsILMiT]2.4 (Stock # 24613 from the Bloomington *Drosophila* Stock Center, Bloomington, IN) as the source of transposase. Finally, for nine genes, we crossed Transgenic RNAi Project (TriP) lines to an *elav*-GAL4 driver to reduce their expression pan-neuronally^84–86^. Supplementary Table S10 lists the TRiP RNAi lines, genotypes, Bloomington stock numbers, and *elav*-GAL4 line used.

### Sleep and activity assays

Previously, sleep and activity was measured in flies from the *Drosophila* Genetic Reference Panel^19^. We briefly describe the original assay conditions here; the full details can be found in^19^. To measure sleep, flies were reared under standard conditions (cornmeal-molasses-agar food medium, 25 °C, 60–75% humidity, and a 12-h:12-h light:dark cycle). Both mating and social enrichment can affect sleep in flies^87,88^, thus virgin males and females were collected and held at 30 flies to a same-sex vial for four days to standardize the impact of these experimental parameters on sleep. We monitored the flies’ sleep and activity in DAM2 *Drosophila* Activity Monitors (Trikinetics, Waltham, MA) for seven days. Flies were allowed to recover from CO_2_ and acclimate to the monitor tubes during the first day, thus data from the first day was removed from further analysis. Data from flies not surviving the assay was also removed from further analysis. For sleep assays, the 167 DGRP lines were split into 4 blocks of 41–42 lines; each block was replicated four times. Each replicate contained 8 flies per sex per line. Eight male and eight female flies of *w*^1118^; *Canton-S B* were measured in each block and replicate as a contemporaneous control. 10,703 flies survived the assay. We used the raw sleep and activity data to calculate the sleep latency, which we defined as the number of minutes to the first sleep bout during the night period. We calculated the sleep latency per fly each day using a C# program (R. Sean Barnes). If the fly was already asleep when the lights were turned off in the incubator, we defined the sleep latency as zero.

For the verification tests, *Minos* lines were subjected to the same procedure, except for the following differences. Standard *Drosophila* medium was used (https://bdsc.indiana.edu/information/recipes/bloomfood/html), and flies were collected and held at 20 flies rather than 30 flies to a same-sex vial for four days. We replicated sleep and activity measures twice. Each replicate contained 8 flies per sex per line, for a total of 16 flies/sex/line tested.

### Quantitative genetic analysis of sleep latency

We used an analysis of variance to partition the variance in sleep latency. The model was

$$Y = \mu + B + S + L\left( B \right) + S \times L\left( B \right) + R\left( B \right) + S \times R\left( B \right) + R \times L\left( B \right) + S \times R \times L\left( B \right) + \varepsilon ,$$

where *B* is the random effect of block, *S* is Sex, *L* is the random effect of DGRP Line, and *R* is the random effect of replicate. We also analyzed the data for sexes separately using the reduced model

$$Y = \mu + B + L\left( B \right) + R\left( B \right) + R \times L\left( B \right) + \varepsilon ,$$

where the terms are defined as above. Broad-sense heritability (*H*^2^) was calculated for both sexes combined as *H*^2^ = *σ*^2^_*L*_ + *σ*^2^_*S*×*L*_/(*σ*^2^_*L*_ + σ^2^_*S*×*L*_ + σ^2^_*E*_) and for sexes separately as *H*^2^ = *σ*^2^_*L*_/( σ^2^_*L*_ + *σ*^2^_*E*_), where σ^2^_*L*_ is the among-line variance, σ^2^_*L*×*S*_ is the line-by-sex variance component, and σ^2^_*E*_ is the sum of all other sources of variance. The cross-sex genetic correlation was computed as *r*_mf_ = *σ*^2^_*L*_/√(*σ*^2^_*LM*_ + *σ*^2^_*LF*_) where *σ*^2^_L_ is the line variance component for both sexes combined, *σ*^2^_*LM*_ is the line variance component for males, and *σ*^2^_*LF*_ is the line variance component for females^89^. In addition, we calculated the genetic correlation between sleep latency and other sleep and circadian traits published previously. The genetic correlation was computed using *r*_G_ = *cov*_12_/√(*σ*_L1_^2^ × *σ*_L2_^2^), where *cov*_12_ is the covariance between traits, and *σ*_L1_^2^ and *σ*_L2_^2^ are the among-line variance estimates for each trait. SAS (SAS Institute, Cary, NC) was used for all statistical analyses.

### Genotype–phenotype associations

We used the DGRP Freeze 2.0 webtool for genome-wide association analysis (dgrp2.gnets.ncsu.edu). This tool tested sleep latency against 1,920,276 genome-wide polymorphisms segregating in the DGRP having minor allele frequencies ≥ 0.05 (9 or more lines having the minor allele)^17^. The webtool first adjusts phenotypes for cryptic genetic relatedness, the presence of chromosomal inversions, and *Wolbachia pipientis* infection status^17^. Neither the inversions nor *Wolbachia* infection status was significantly associated with sleep latency; however, adjustments were made to the sleep latency phenotype based on cryptic relatedness (Supplementary Table S1). Using the adjusted data, we then applied the following linear mixed model:

$$y = Xb + Zu + e$$

where *y* is the adjusted sleep latency means, *X* is the design matrix containing the fixed effect of each SNP, *Z* is the incidence matrix of random effects, and *e* is the random residual error^17^.

We defined our threshold *P*-value for each association as *P* ≤ 1 × 10^–5^, a threshold used in many other studies of the DGRP^22,37,41,90–94^ and supported by Q-Q plots (Supplementary Fig. S1). Variants meeting the threshold criterion were mapped to the *Drosophila* 6.0 genome. We used the DRSC Integrative Ortholog Prediction Tool (DIOPT) to identify *Drosophila* genes having human homologs^27^, and the Database for Annotation, Visualization, and Integrated Discovery (DAVID) to assess gene ontology and pathway enrichment^27^.

### Pairwise epistasis and network analysis

We tested all significant polymorphisms with a minor allele frequency of 0.15 or greater (43 polymorphisms; 903 total pairwise tests) for possible pairwise epistatic interactions. Pairs were tested for high (*r*^2^ ≥ 0.8) linkage disequilibrium (LD) using PLINK^95^; none of the polymorphisms were in high LD. We applied the following model for males and females separately:

$$Y = \mu + M1 + M2 + M1 \times M2 + \varepsilon$$

where *M1* is the first marker tested, *M2* is the second marker tested, and ε is error. We used a Bonferroni-corrected *P*-value to identify potential interactions (i.e., a significant *M*1 × *M*2 term).

In addition, we used BIOGRID to identify known genetic and protein–protein interactions among sleep latency candidate genes^29^. We visualized these interactions using Cytoscape^96^.

### Candidate gene verification

To identify candidate genes for further testing we developed the following criteria: the polymorphism (1) must be located within ± 1000 bp of a gene; (2) within the top 100 polymorphisms with the largest predicted effect size for sexes combined; and (3) have a *Minos* insertion stock available for testing. We found 23 such genes (Supplementary Table S9). We measured the sleep latency in each mutant and compared it to its corresponding isogenic control line (*w*^1118^ or *y*^1^*w*^67c23^). We measured 8 flies per sex per line in two separate biological replicates. We analyzed the data using the ANOVA model

$$Y = \mu + G + S + R + G \times S + G \times R + R \times S + G \times S \times R + \varepsilon$$

where *µ* is the overall population mean, *G* is genotype, *S* is Sex, *R* is replicate, and *ε* is the within-line variance. We used a Bonferroni-corrected *P*-value of 0.0031 as our criterion for significance for lines having the *w*^1118^ control (16 lines) and 0.0071 as the criterion for lines having the *y*^1^*w*^67c23^ control (7 lines). We additionally tested for differences among the mutants without the control lines using a Dunnett’s *t*-test.

We additionally used available RNAi lines to knock down gene expression in nine of the genes having significant *Minos* phenotypes. We used Transgenic RNAi Project (TriP) lines as the source of RNAi hairpins (Supplementary Table S10)^84,85^. We used an *elav*-GAL4 driver to reduce gene expression pan-neuronally^86^. The *elav*-GAL4 driver was crossed to each RNAi line, and sleep and activity were measured and analyzed as stated above. We used a Bonferroni-corrected *P*-value of 0.0056 as our criterion for significance. In addition, we tested whether sleep latency in the *elav*-GAL4 × RNAi cross progeny was different from what would be expected under a strict additive model (i.e., the mean of the sleep latency for the *elav*-GAL4 and corresponding RNAi line) using a t-test.

### Genetic rescue of *Piezo* and *Proc-R*

We used a previously published procedure to produce precise excision lines from *Piezo* and *Proc-R Minos* insertion lines^30^. We crossed flies from a helper line, *w*^1118^; *sna*^Sco^/SM6a, *P*[*w*[+ mC] = hsILMiT]2.4 to flies homozygous for the *Mi*[*ET1*] insertion in *Piezo* and in *Proc-R*. After two days, the parental flies were removed. We exposed larval progeny to heat shock using a 37 °C water bath for 1 h per day for 3 days. After eclosion, the adult progeny were screened for the presence of mosaic green fluorescent protein (GFP) in the eyes, an indication that the *Minos* element has been partially transposed. Individual mosaic males were backcrossed to females from their respective *Minos* insertion line as appropriate (*Piezo*^MB08675^ or *Proc-R*^MB00909^). The resulting heterozygous progeny were crossed and screened for the presence of GFP. GFP-negative flies were crossed to produce precise excision lines, *Piezo*^MB*^ and *Proc-R*^MB*^. We verified precise excisions via DNA extraction with PCR amplification and sequencing.

PCR reactions were carried out in 0.2 mL PCR tube strips with strips of dome caps (USA Scientific, Inc., Ocala, FL) using Qiagen *Taq* PCR Master Mix containing 250 units *Taq* DNA Polymerase (Cat. No./ID: 201443 Qiagen). The PCR reactions were performed on an Eppendorf Mastercycler nexus PCR cycler (Eppendorf North America, Enfield, CT) using the manufacturer’s protocol. We designed primers using Primer-Blast (https://www.ncbi.nlm.nih.gov/tools/primer-blast/). Primers were synthesized by IDT (Integrated DNA Technologies, Coralville, IA).

Primers to amplify and sequence a region of *Piezo* flanking the *Minos* insertion site in *w*^*1118*^ and *Piezo*^MB*^ DNA were 1, *TCCTGACCGAGGGATTTTTGG* and 2, *ACACAAGTGAATCCCCTGAAGC*. To confirm the location of the *Minos* element *Mi*[*ET1*] in *Piezo*^*MB08675*^, a fragment spanning the junction between *Piezo* and *Mi*[*ET1*] was amplified and sequenced using primers 7, *CCGAGGGATTTTTGGAACCG* and 8, *CTCATGTTTGACAGCTTATCATCG*. A region flanking the *Minos* insertion site in *Proc-R* in *w*^*1118*^ and *Proc-R*^MB*^ DNA was amplified using primers 20, *TGTTCAGTATTTCCGCTACATTTGC* and 21, *TGATCTTATCTCCGTACGCTGC*. The *Proc-R* fragments were sequenced using primers 30, *GGAGAAAATTAAACTGCTCGGC*, 32, *TCATGAGATACAAAATGGCGGG*, and 34, *GGCGGGGAGATGAAGATTTTGG*. The location of *Mi*[*ET1*] in *Proc-R*^*MB00909*^ was confirmed using primers 22, *GATGGACTTGCTGCCATGACC*, 23, *CATGCTGGAGTTCTTCGCCC*, and 30. See Supplementary Fig. S3 for a schematic of *Minos* element insertion and primer locations as well as the gel showing that the PCR fragments were of the expected size. Supplementary Fig. S4 presents confirmatory sequence data.

We tested the putative precise excision lines for sleep latency and compared them to the *w*^1118^ isogenic control, the *Piezo*^MB08675^ (*Proc-R*^MB00909^) *Minos* insertion line, as appropriate, and a heterozygous cross between *w*^1118^ and *Piezo*^MB08675^ (*Proc-R*^MB00909^). We measured 16 flies per sex per line, and the experiments were replicated twice. The same ANOVA model used for the verification tests was used for the rescue experiments, along with post-hoc Tukey tests to distinguish differences among individual genotypes.

### Arousal threshold measures in *Piezo* mutant and rescue lines

We assessed the arousal threshold in the *Piezo*^MB08675^ *Minos* insertion line, the *Piezo*^MB*^ rescue line, and the *w*^1118^ control. We perturbed male and female flies from each line for one second per hour for three days using the Trikinetics Vortexer mounting plate system (Trikinetics, Waltham, MA). The vortexer was set to the lowest setting, intensity 1. For each fly at each hour, we computed the amount of sleep in minutes prior to the stimulus and the number of minutes the fly was awake after the stimulus. We also calculated the proportion of flies responding to the stimulus each hour^97,98^. We analyzed the data using the ANOVA model *Y* = *µ* + *G* + *S* + *G* × *S* + *ε*, where G and S are as defined above.

### Supplementary Information

Supplementary Figures.

Supplementary Tables.

## Data Availability

The data generated for this manuscript are provided in the supplementary tables. We downloaded publicly available genome variant data for the DGRP from the DGRP2 website, dgrp2.gnets.ncsu.edu. Additional information on the DGRP, including the SRA accession numbers for the DNA sequences of each DGRP line, can be found in Huang et al.^17^.
