# Supplementary material for: Genome-wide association in Drosophila identifies a role for Piezo and Proc-R in sleep latency
Source: Sci Rep. 2024 Jan 2;14:260. doi: 10.1038/s41598-023-50552-z (PMC10761942; doi:10.1038/s41598-023-50552-z)
Supplement: Supplementary file 1 — Supplementary Figures. [file 41598_2023_50552_MOESM1_ESM.pdf]

Genome-wide association in *Drosophila* identifies a role for *Piezo* and *Proc-R* in sleep latency

Matthew N. Eiman, Shailesh Kumar, Yazmin L. Serrano Negron, Terry R. Tansey, and Susan T. Harbison

Supplemental Figures S1 – S5

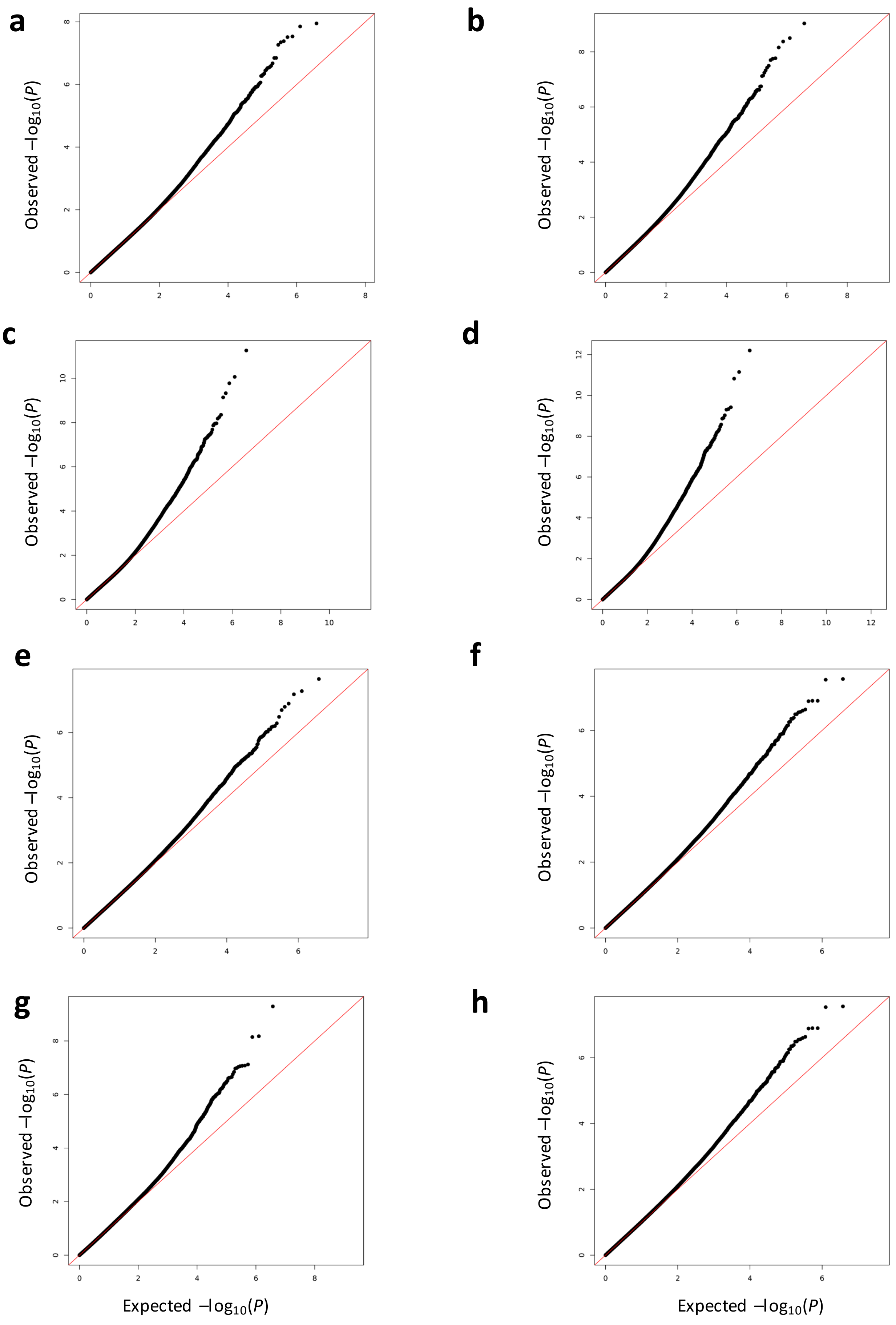
a
b
c
d
e
f
g
h
Observed −log10(P)
Expected −log10(P)

Figure S1. Q-Q plots for sleep latency. The plots show the observed versus the expected *P*-value distribution, plotted as the -log(*P*-value). (**a**) and (**b**), sleep latency for sexes combined using the (**a**) mixed model adjusting for cryptic relatedness and (**b**) using the unadjusted model. (**c**) and (**d**) sleep latency for females using the (**c**) mixed model adjusting for cryptic relatedness and (**d**) using the unadjusted model. (**e**) and (**f**), sleep latency for males using the (**e**) mixed model adjusting for cryptic relatedness and (**f**) using the unadjusted model. (**g**) and (**h**) sleep latency sex difference (male – female) using the (**g**) mixed model adjusting for cryptic relatedness and (**h**) using the unadjusted model.

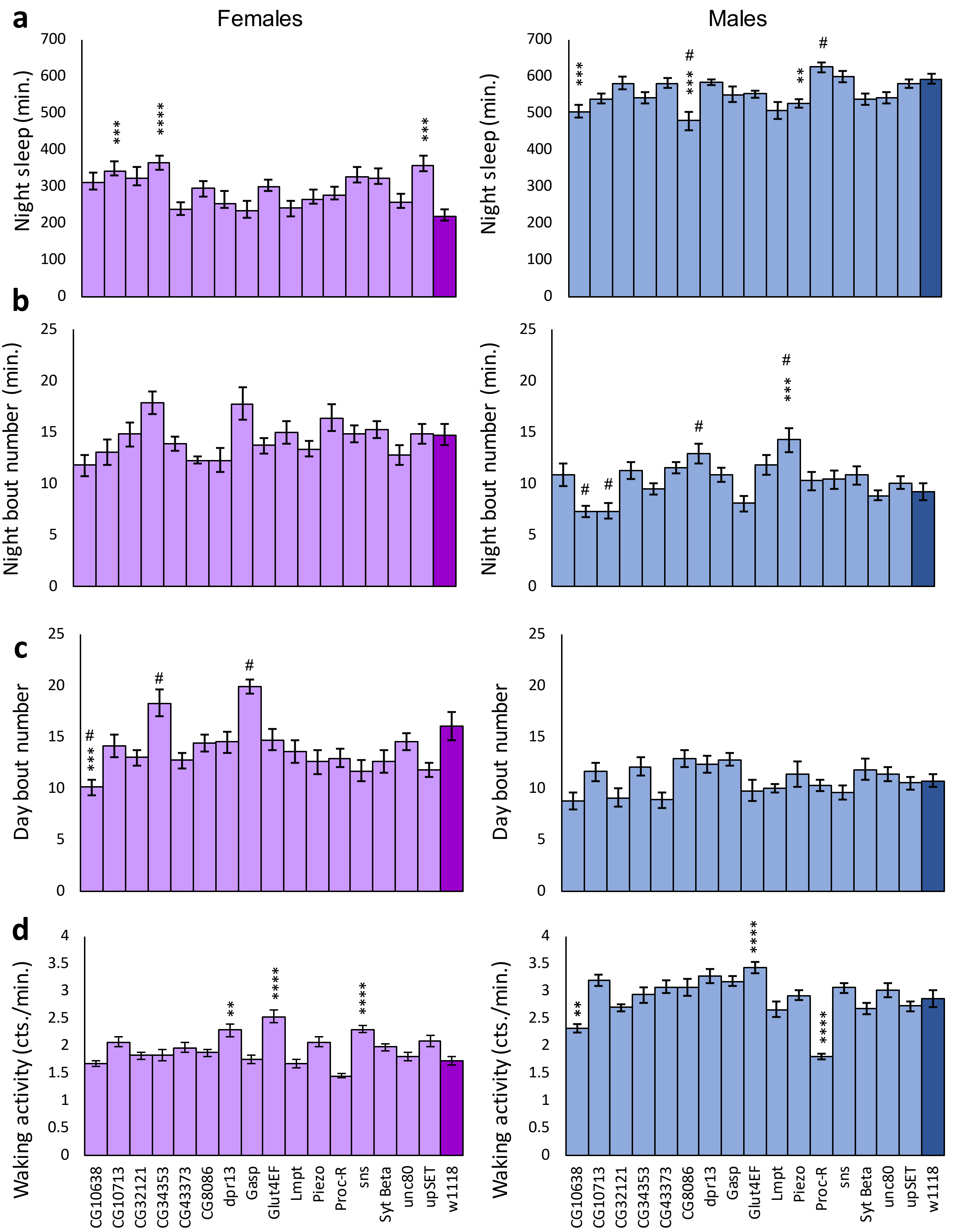

a
Females
Males
Night sleep (min.)
b
Night bout number (min.)
c
Day bout number
d
Waking activity (cts./min.)
CG10638
CG10713
CG32121
CG34353
CG43373
CG8086
dpr13
Gasp
Glut4EF
Lmpt
Piezo
Proc-R
sns
Syt Beta
unc80
upSET
w1118

Figure S2. ***Minos*** **insertion line phenotypes with little pleiotropy (*w*^{1118} control).** The plots show mean sleep phenotypes ± SEM in *Minos* insertion lines contrasted with the sleep phenotypes of the *w*^{1118} isogenic control. Light purple bars indicate female data, with the *w*^{1118} control shown in dark purple. Light blue bars indicate male data, with the *w*^{1118} control shown in dark blue. (**a**), night sleep duration. (**b**), night bout number. (**c**), day bout number. (**d**), waking activity. Asterisks show the level of significance: **** $P \le 0.0001$; *** $0.0001 < P \le 0.001$; ** $0.001 < P \le 0.0031$. A pound sign (#) shows those mutants that are also significantly different from the overall mean of all mutant data combined.

a

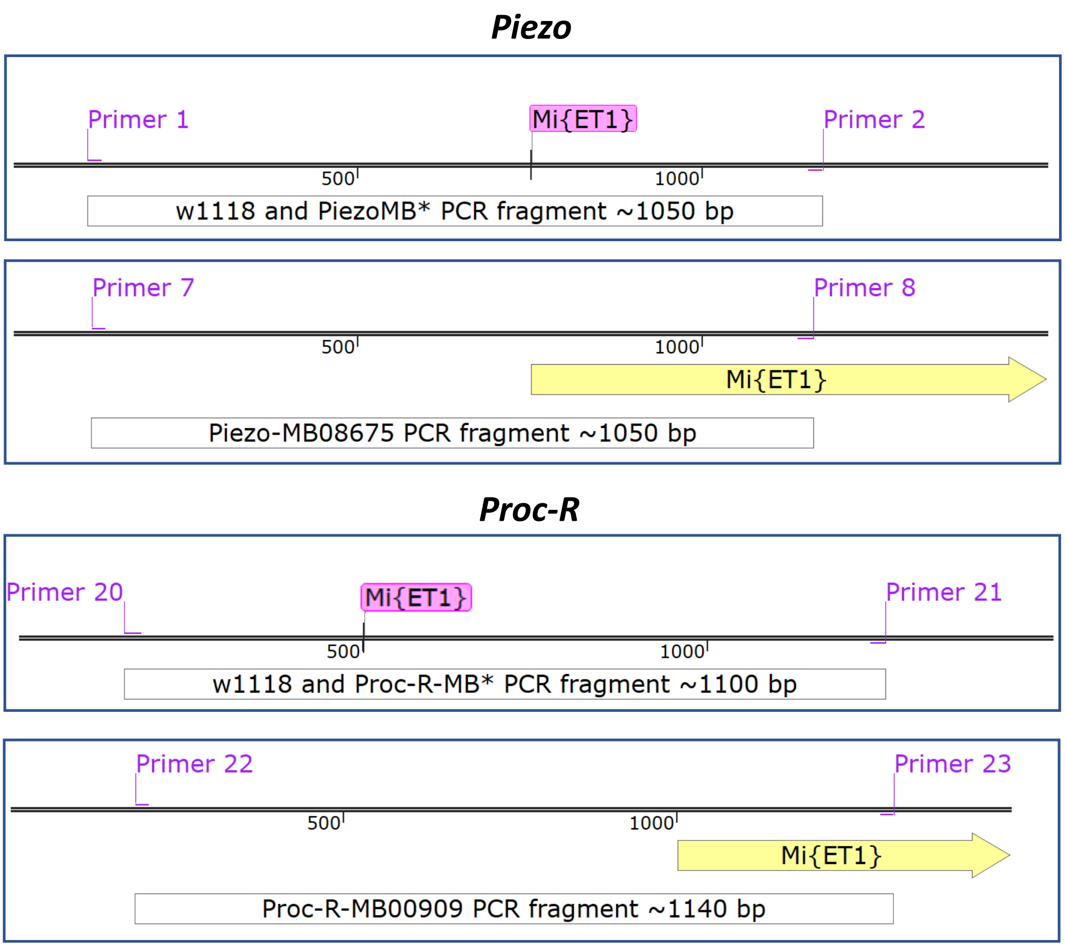

Piezo
Primer 1
Mi{ET1}
Primer 2
500
1000
w1118 and PiezoMB* PCR fragment ~1050 bp
Primer 7
Primer 8
500
1000
Mi{ET1}
Piezo-MB08675 PCR fragment ~1050 bp
Proc-R
Primer 20
Mi{ET1}
Primer 21
500
1000
w1118 and Proc-R-MB* PCR fragment ~1100 bp
Primer 22
Primer 23
500
1000
Mi{ET1}
Proc-R-MB00909 PCR fragment ~1140 bp

b

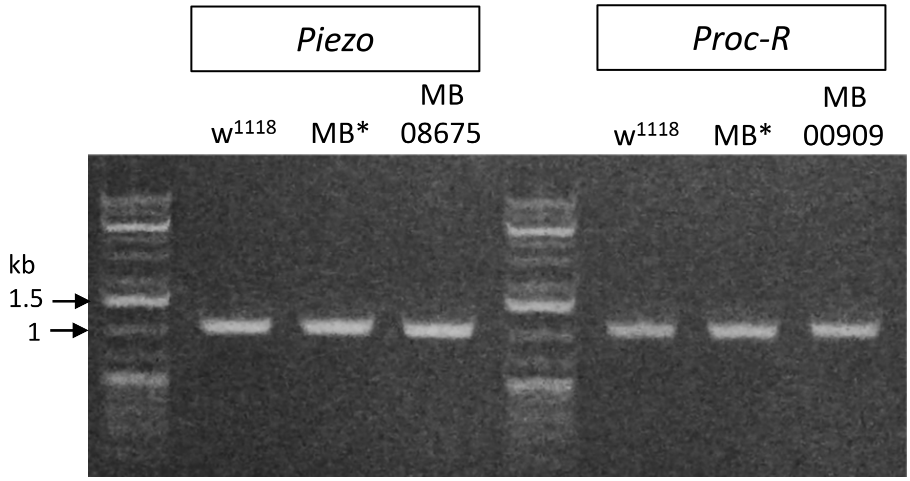

Piezo
Proc-R
w1118
MB*
MB 08675
w1118
MB*
MB 00909
kb
1.5
1

c

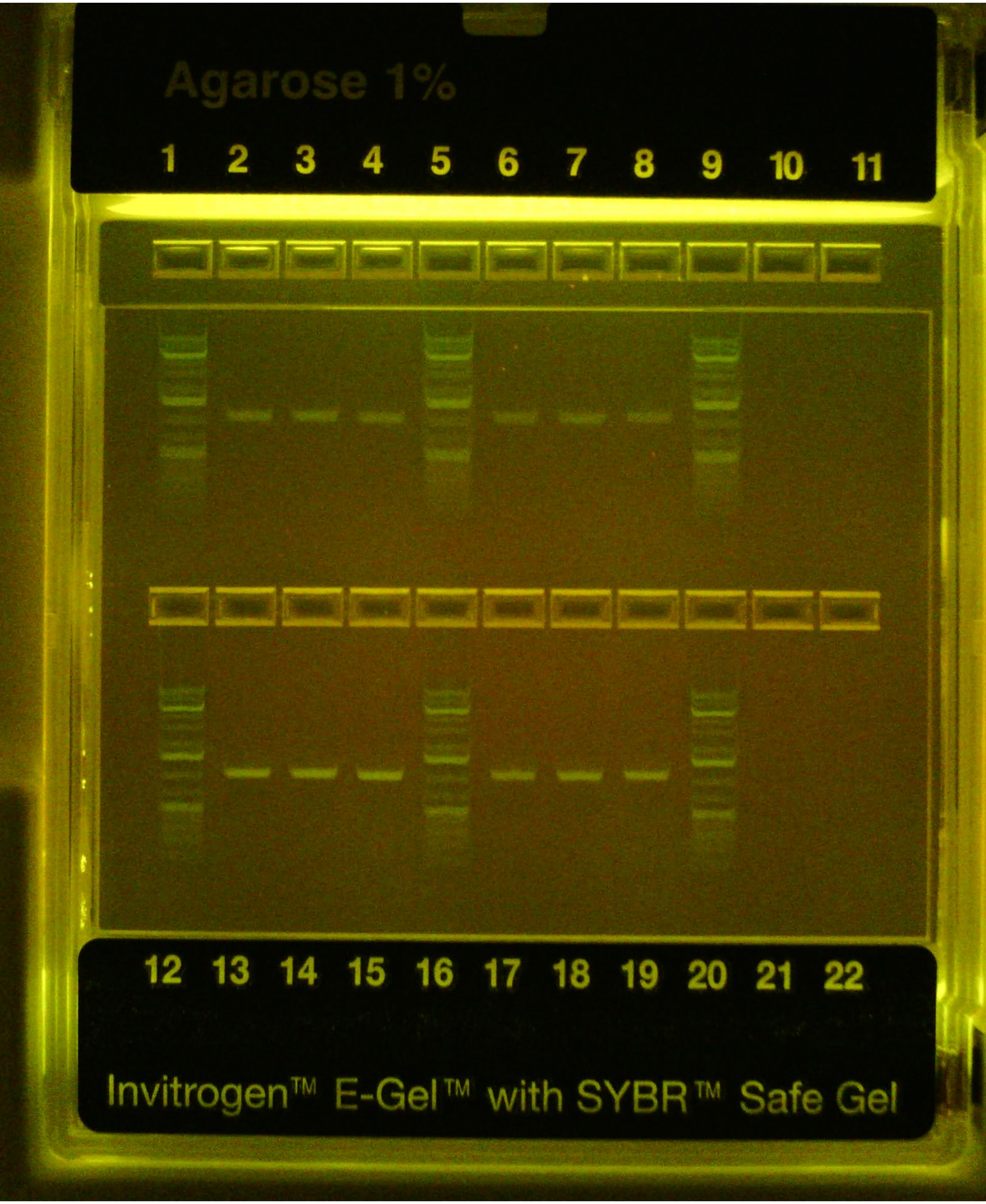

Agarose 1%
1 2 3 4 5 6 7 8 9 10 11
12 13 14 15 16 17 18 19 20 21 22
Invitrogen™ E-Gel™ with SYBR™ Safe Gel

Gel lane key

Lane ID	1	2	3	4	5	6	7	8	9	10	11
Sample ID	Ladder	w^{1118}	*Piezo* MB*	*Piezo* MB^{08675}	Ladder	w^{1118}	*Proc-R* MB*	*Proc-R* MB^{00909}	Ladder	Empty	Empty
Lane ID	12	13	14	15	16	17	18	19	20	21	22
Sample ID	Ladder	w^{1118}	*Piezo* MB*	*Piezo* MB^{0867}	Ladder	w^{1118}	*Proc-R* MB*	*Proc-R* MB^{00909}	Ladder	Empty	Empty

Figure S3. Schematic and gel for precise excision of *Piezo* and *Proc-R Minos* elements. PCR fragments used to confirm the sequence of *Piezo* and *Proc-R* alleles. (**a**) Diagrams of the locations of the PCR fragments in relation to the *Minos* insertion sites. (**b**) Agarose gel of the PCR fragments used for sequencing, indicating that the fragments were of the expected size. Gel was cropped and converted to grayscale; brightness was adjusted +20; and contrast was adjusted +40 in Excel. (**c**) Full-length unadjusted gel from (**b**) with gel lane key diagram showing the samples that were loaded in each lane. Orange shading shows lanes loaded with 50 ng of sample; gray shading shows lanes loaded with 100 ng of sample.

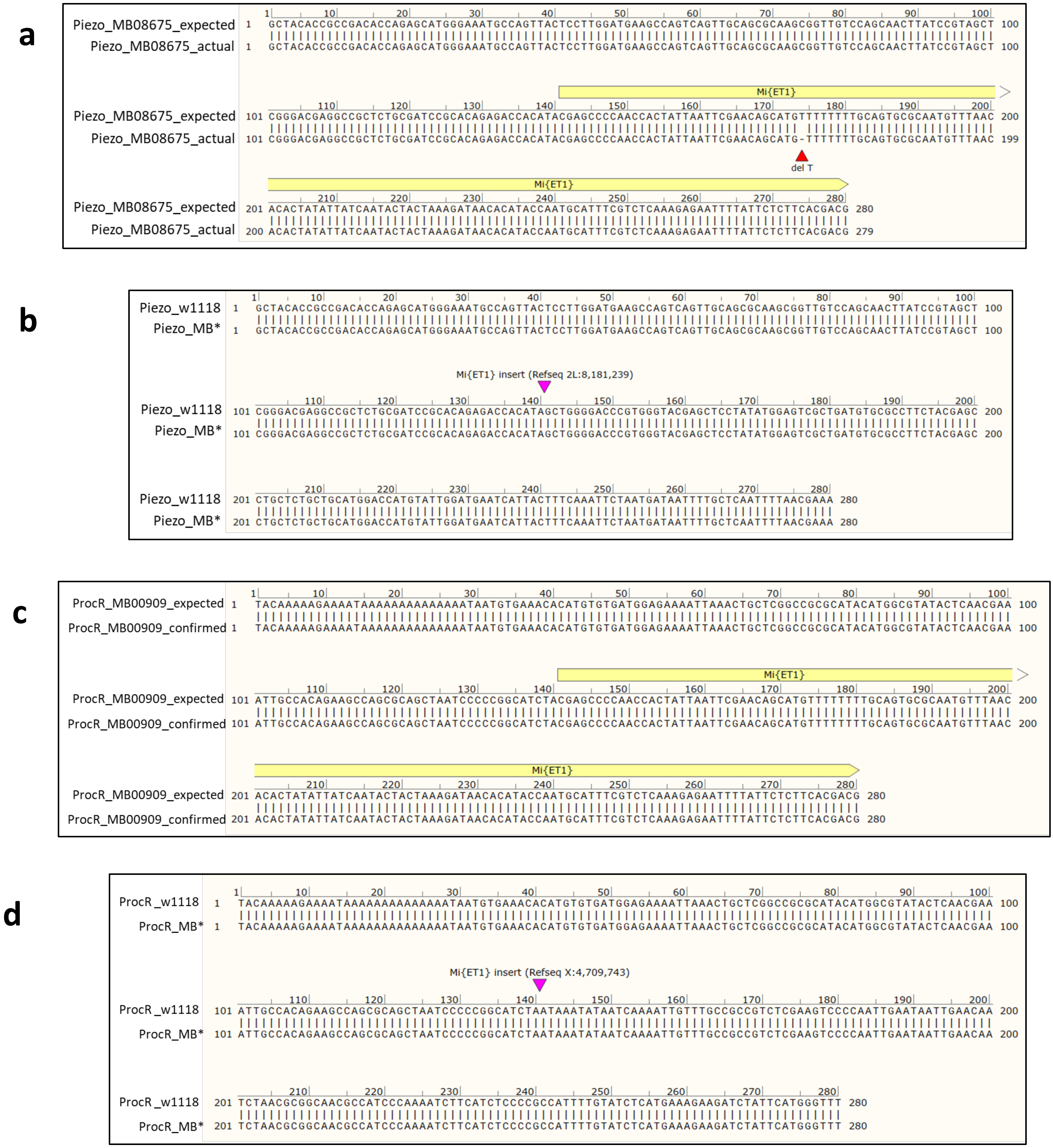

Figure S4. Aligned confirmatory sequences. (**a**) The expected location of the *Minos* element Mi[ET1] in *Piezo*^{MB08675}. Mi[ET1]*Piezo*^{MB08675} has a single nucleotide deletion (at red arrowhead) compared to the expected sequence. Although FlyBase indicates that Mi[ET1]*Piezo*^{MB08675} is inserted at *2L*:8181238, sequencing in the present study places the insertion at *2L*:8181239. (**b**) Precise excision of the *Minos* element in *Piezo*^{MB*}. (**c**) The expected location of the *Minos* element Mi[ET1] in *Proc-R*^{MB00909}. (**d**) Precise excision of the *Minos* element in *Proc-R*^{MB*}.

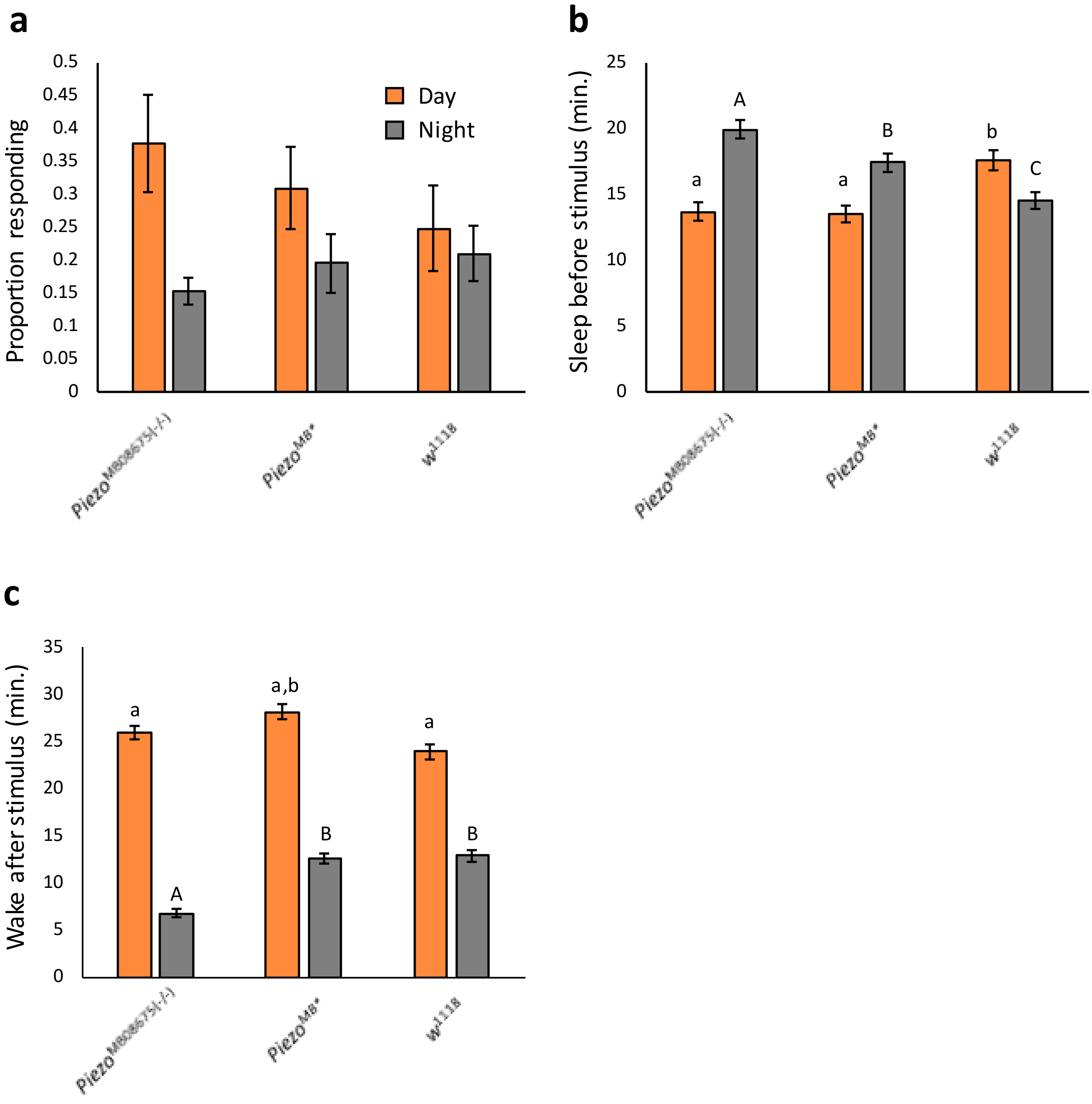

Figure S5. ***Piezo*** **mutants have an increased propensity to sleep during the night.** (**a**), The proportion of flies responding to a mechanical stimulus. (**b**), The amount of time spent sleeping before the mechanical stimulus. (**c**), The amount of time spent awake after the mechanical stimulus.
